# Association with Controlling Nutritional Status (CONUT) Score and In-hospital Mortality and Infection in Acute Heart Failure

**DOI:** 10.1038/s41598-020-60404-9

**Published:** 2020-02-24

**Authors:** Takao Kato, Hidenori Yaku, Takeshi Morimoto, Yasutaka Inuzuka, Yodo Tamaki, Erika Yamamoto, Yusuke Yoshikawa, Takeshi Kitai, Ryoji Taniguchi, Moritake Iguchi, Masashi Kato, Mamoru Takahashi, Toshikazu Jinnai, Tomoyuki Ikeda, Kazuya Nagao, Takafumi Kawai, Akihiro Komasa, Ryusuke Nishikawa, Yuichi Kawase, Takashi Morinaga, Kanae Su, Mitsunori Kawato, Yuta Seko, Moriaki Inoko, Mamoru Toyofuku, Yutaka Furukawa, Yoshihisa Nakagawa, Kenji Ando, Kazushige Kadota, Satoshi Shizuta, Koh Ono, Yukihito Sato, Koichiro Kuwahara, Neiko Ozasa, Takeshi Kimura

**Affiliations:** 1 Department of Cardiovascular Medicine, Kyoto University Graduate School of Medicine, Kyoto, Japan; 2 Department of Clinical Epidemiology, Hyogo College of Medicine, Hyogo, Japan; 3 Department of Cardiovascular Medicine, Shiga Medical Center for Adult, Shiga, Japan; 4 Division of Cardiology, Tenri Hospital, Nara, Japan; 5 Department of Cardiovascular Medicine, Kobe City Medical Center General Hospital, Hyogo, Japan; 6 Department of Cardiology, Hyogo Prefectural Amagasaki General Medical Center, Hyogo, Japan; 7 National Hospital Organization Kyoto Medical Center, Kyoto, Japan; 8 Mitsubishi Kyoto Hospital, Kyoto, Japan; 9 Shimabara Hospital, Kyoto, Japan; 10 Japanese Red Cross Otsu Hospital, Shiga, Japan; 11 Hikone Municipal Hospital, Shiga, Japan; 12 Osaka Red Cross Hospital, Osaka, Japan; 13 Kishiwada City Hospital, Osaka, Japan; 14 Kansai Electric Power Hospital, Osaka, Japan; 15 Shizuoka General Hospital, Shizuoka, Japan; 16 Kurashiki Central Hospital, Okayama, Japan; 17 Kokura Memorial Hospital, Fukuoka, Japan; 18 Japanese Red Cross Wakayama Medical Center, Wakayama, Japan; 19 Nishikobe Medical Center, Hyogo, Japan; 20 Kitano Hospital, Osaka, Japan; 21 Department of Cardiovascular Medicine, Shinshu University Graduate School of Medicine, Matsumoto, Japan

**Keywords:** Prognostic markers, Heart failure, Malnutrition

## Abstract

The high controlling nutritional status (CONUT) score that represents poor nutritional status has been acknowledged to have prognostic implications in chronic heart failure. We aimed to investigate its role in acute decompensated heart failure (ADHF). Using the data from an multicenter registry that enrolled 4056 consecutive patients hospitalized for ADHF in Japan between 2014 and 2016, we analyzed 2466 patients in whom data on the components of the CONUT score at hospital presentation were available. The decrease of lymphocyte count and total cholesterol was assigned with 0, 1, 2, and 3 points and the decrease of albumin was assigned with 0, 2, 4, and 6 points according to the severity. We defined low CONUT score as 0–4 (N = 1568) and high CONUT score as 5–9 (N = 898). The patients in the high CONUT score group were older and more likely to have a smaller body mass index than those in the low CONUT score group. The high CONUT score group was associated with higher rate of death and infection during the index hospitalization compared to the low CONUT score group (9.0% versus 4.4%, and 21.9% versus 12.7%, respectively). After adjusting for confounders, the excess risk of high relative to low CONUT score for mortality and infection was significant (OR: 1.61, 95%CI: 1.05–2.44, and OR: 1.66, 95%CI: 1.30–2.12, respectively). The effect was incremental according to the score. High CONUT score was associated with higher risk for in-hospital mortality and infection in an incremental manner in patients hospitalized for ADHF.

## Introduction

Despite recent advances in chronic heart failure (HF) therapy, there remain unmet needs to reduce the high mortality rate and to assess the mortality risk in patients hospitalized for acute decompensated HF (ADHF)^1^. Recently, there was a line of evidences that nutritional status has a link to the prognosis of patients with chronic HF^2–4^. Malnutrition in HF is related to altered intestinal function^5^, which could be due to hemodynamic changes. Intestinal congestion leads to bowel edema and overgrowth of bacteria flora, resulting in malabsorption, chronic inflammation, and malnutrition^6,7^. The pathophysiology of cardiac malnutrition involves a catabolic wasting state associated with inflammation and coincident neurohormonal activation frequently observed in HF^8^. However, the setting of ADHF is more complex. ADHF is considered to be an acute condition in which symptoms occur de novo or chronic HF exaggerates. In ADHF, the patients were hemodynamically unstable with marked congestion, and presented with concurrent anemia and renal failure^9^. The sympathetic nervous system and the neurohormonal axis including the renin angiotensin aldosterone system in ADHF were markedly stimulated^10^. Thus, acute condition may modify the baseline nutritional status in patients with ADHF.

The Controlling Nutritional Status (CONUT) score is a screening tool to identify undernourished patients in the hospitalized population^11^. The score is derived from the values of serum albumin, total cholesterol and lymphocyte counts. Albumin represents the protein reserves; total cholesterol represents caloric depletion; and lymphocyte count represents immune defense^11^. The decrease in each component was assigned with high score. Thus, the higher score means the worse nutritional status. Although it was originally developed to predict acute worsening in surgical patients, high CONUT score has been known to have a prognostic impact in patients with chronic cardiac disease^5,12–14^. These findings were mainly derived from the study of stable outpatient with HF with reduced ejection fraction. Given the CONUT score includes the nutrition and immune response, we hypothesized that the scores at hospital presentation can predict in-hospital prognosis of the patients with ADHF independently of other known prognostic factors.

## Methods

### Study population

**Figure 1:**
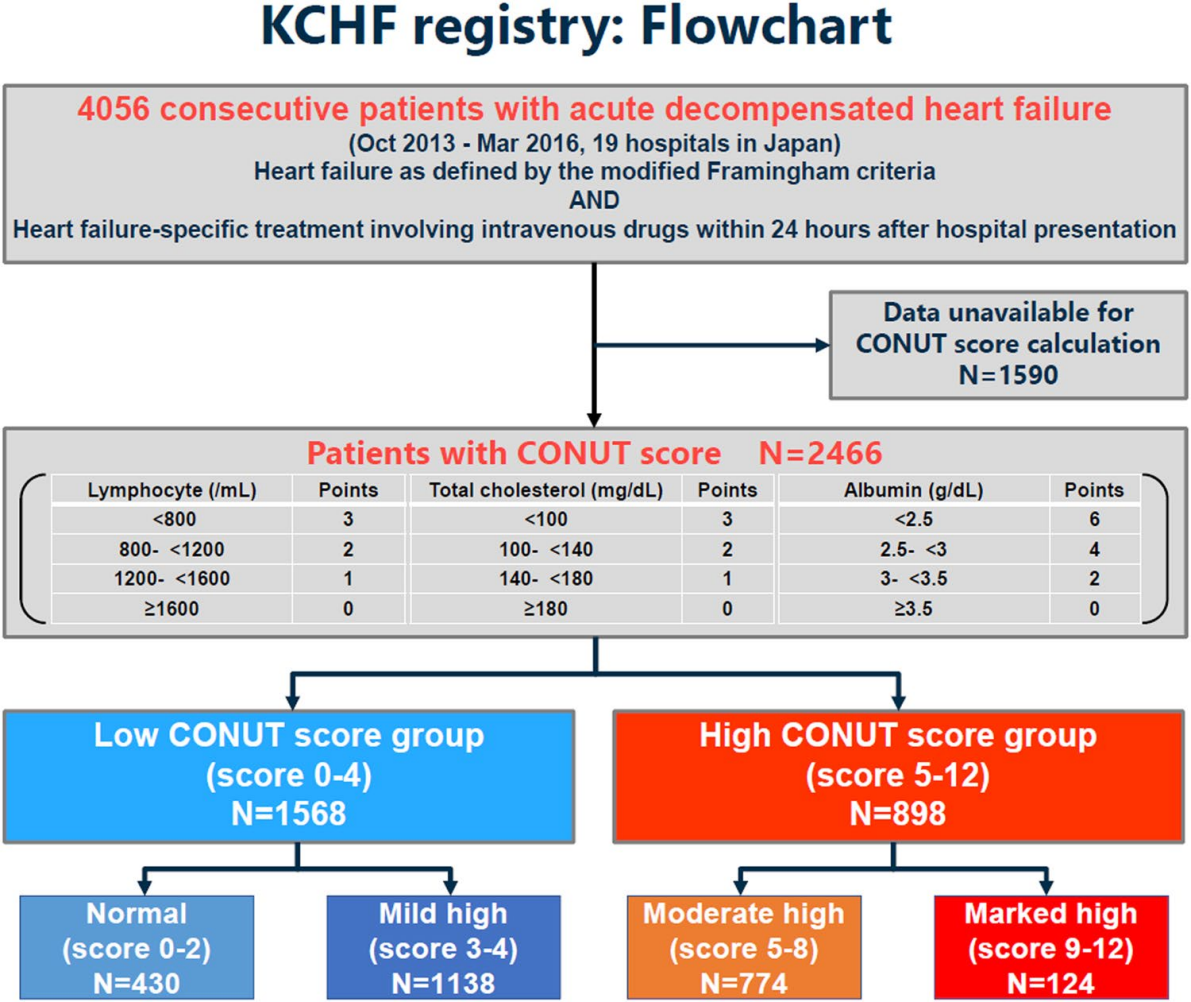
Patient flowchart. KCHF = Kyoto Congestive Heart Failure, CONUT = Controlling Nutritional Status.

**Figure 2:**
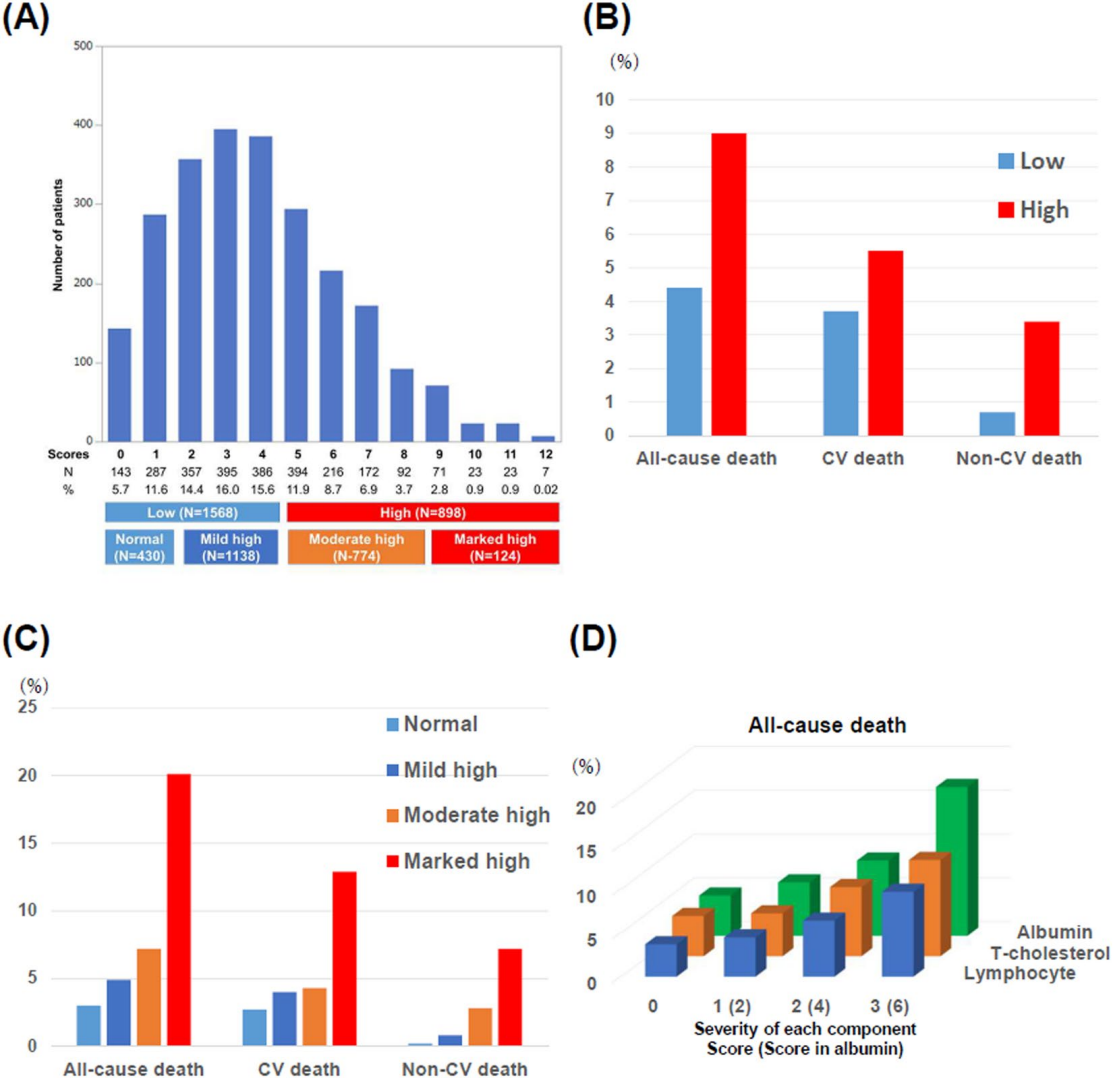
(**A**) Distribution and classification of the CONUT scores. CONUT = Controlling Nutritional Status (**B**) Crude incidence of all-cause, cardiovascular, and non-cardiovascular deaths in the low and high CONUT score groups. CV = cardiovascular. (**C**) Crude incidence of all-cause, cardiovascular, and non-cardiovascular deaths when classified into 4 groups: normal, mild high, moderate high, and marked high groups. (**D**) Crude incidence of all-cause according to the severity of each component of CONUT score. T-Cholesterol = Total cholesterol.

The Kyoto Congestive Heart Failure (KCHF) registry is a physician-initiated, prospective, observational, multicenter cohort study that enrolled, without exclusion, consecutive patients who were hospitalized for ADHF for the first time between October 2014 and March 2016 in the 19 participating hospitals in Japan. The overall design of the KCHF study has been previously described in detail^15^. We enrolled consecutive patients with ADHF as defined by the modified Framingham criteria admitted to the participating centers, who underwent heart failure-specific treatment involving intravenous drugs within 24 hours after hospital presentation. Among the 4056 patients enrolled in the registry, the present study population consisted of 2466 patients whose CONUT score had been calculated at the time of hospital presentation, after excluding 1590 patients with missing data for calculation of their CONUT scores (Fig. 1). Number of patients with missing values for each component of the CONUT score was provided in Supplementary Table 1. CONUT score was calculated according to the original study^11^, as shown in Supplementary Table 2. We defined low CONUT score as 0–4 and high CONUT score as 5–12 according to the median values of 4 in this cohort (Fig. 2). In the present analysis, we compared the baseline characteristics and in-hospital outcomes between the high CONUT score group (N = 898) and low CONUT score group (N = 1568) (Figs. 1 and 2A).

### Ethics

The investigation conforms with the principles outlined in the Declaration of Helsinki. The study protocol was approved by the ethical committees at the Kyoto University Hospital (local identifier: E2311) and each participating hospital (Supplementary Materials). The requirement for written informed consent from each patient was waived because of the following reasons^9,15^, which were in accordance with Japanese ethical guidelines for epidemiologic research^16^: (1) we would use clinical information obtained in routine practice on the medical record without any risk to the subjects, (2) the waiver of normal consent procedures would not affect adversely the rights and welfare of the subjects, (3) the research could not be carried out effectively without the waiver, and (4) the subjects were provided with additional pertinent information and had the right to opt out of this study whenever appropriate. This study was registered with UMIN (UMIN identifier: UMIN000015238.

### Data collection and definitions

We collected data on patient demographics, medical history, underlying heart disease, pre-hospital activities, socioeconomic status, signs, symptoms, medications, laboratory tests at hospital presentation, electrocardiogram, echocardiography, and clinical events during the index hospitalization^9,17,18^.

HF was classified according to left ventricular ejection fraction (LVEF), as HF with preserved LVEF (LVEF ≥ 50%), HF with mid-range LVEF (40%≤ LVEF < 50%), and HF with reduced LVEF (LVEF < 40%)^19^. Other definitions for the baseline factors and calculation formula for geriatric nutritional risk index (GNRI) are provided in the Supplement^9,17,18^.

The primary outcome measure for the present analysis was all-cause death during the index HF hospitalization. The secondary outcome measures included cardiovascular death and non-cardiovascular death, and infection during hospitalization^15^. We set the occurrence of new infection or infection that became obvious after hospitalization as one of the secondary outcomes. The causes of death were classified according to the VARC (Valve Academic Research Consortium) definitions^20^, and were adjudicated by a clinical event committee^9,15,17,18^.

### Statistical analyses

The categorical variables are presented as numbers and percentages, and were compared using a chi-square test or Fisher’s exact test. The continuous variables are expressed as mean (standard deviation [SD]) or median (interquartile range [IQR]). On the basis of their distributions, the continuous variables were compared using Student’s t-test or the Wilcoxon rank sum test between two groups. To estimate the risk of high CONUT relative to low CONUT for the primary and secondary outcome measures, we used a multivariable logistic regression model not accounting for the time to events due to the evaluation of events during the index hospitalization. We included the pre-specified following potential clinically relevant 20 risk-adjusting variables into the model: age (as continuous variables), sex, body mass index (BMI) below 22 kg/m2, LVEF < 40% by echocardiography, acute coronary syndrome as an etiology of heart failure, past history, and presence of comorbidities, as shown in Table 1. In addition, we added 5 risk-adjusting variables: liver cirrhosis and medications at presentation, thus we used 25 variables for adjustment. The adjusted odds ratios (ORs) and 95% confidence intervals (CIs) were calculated. Subgroup analyses for the primary outcome measure were also performed based on LVEF < 40% based on the HF guideline of LVEF classification [19], age 80 years or more based on the median value, sex, BMI < 22 kg/m2, anemia, renal dysfunction (eGFR <30 ml/min/1.73m2) based on CKD grade, C-reactive protein (CRP) levels (CRP > 1 mg/dL) based on cut-off value in metabolic syndrome^21^, and the presence of dyslipidemia. In the subgroup analysis, we used 25 risk-adjusting variables, without adjustment for multiple tests. We also evaluated the interactions between the subgroup factors and the effect of high CONUT score relative to low CONUT score for the primary and secondary outcomes.

**Table 1:** Patient Characteristics of the Study Population.

Variables	CONUT score		
	Low (N = 1568, 63.5%)	High (N = 898, 36.4%)	P value
Clinical characteristics			
Age, years*	79 [70–85]	82 [74–87]	<0.0001
Age >80 years	713 (45.7)	530 (59.0)	<0.0001
Men*	881 (56.1)	531 (59.3)	0.13
BMI ||< 22 kg/m^2^*	607 (40.5)	442 (51.8)	<0.0001
Etiology			
Dilated cardiomyopathy	193 (12.3)	78 (8.6)	0.0056
ACS*	120 (7.6)	50 (5.5)	0.049
Aortic stenosis	111 (7.0)	66 (7.3)	0.80
Hypertensive	401 (25.5)	212 (23.1)	0.27
Ischemic (not acute)	399 (25.4)	230 (25.6)	0.92
Medical history			
Prior hospitalization for HF*	533 (34.4)	338 (38.1)	0.067
Atrial fibrillation or flutter*	615 (39.2)	394 (43.8)	0.023
Hypertension*	1148 (73.2)	616 (68.6)	0.014
Diabetes mellitus*	570 (36.3)	333 (37.0)	0.71
Dyslipidemia	661 (42.1)	323 (35.9)	0.0025
Prior myocardial infarction*	367 (23.4)	192 (21.3)	0.24
Prior stroke*	254 (16.2)	150 (16.7)	0.74
Prior PCI or CABG	407 (25.9)	218 (24.8)	0.35
Current smoking*	233 (14.8)	87 (9.7)	0.0002
Ventricular tachycardia/fibrillation	62 (3.9)	37 (4.1)	0.83
Chronic kidney disease	626 (39.2)	437 (41.1)	<0.0001
Chronic lung disease*	133 (8.4)	83 (9.2)	0.52
Liver cirrhosis**	15 (0.9)	28 (3.1)	<0.0001
Malignancy	209 (13.3)	147 (16.3)	0.038
Dementia	253 (16.1)	236 (25.1)	<0.0001
Social backgrounds			
Poor medical adherence	259 (16.5)	133 (14.8)	0.26
Living alone*	367 (23.5)	172 (19.5)	0.014
With occupation	255 (16.2)	88 (9.8)	<0.0001
Public financial assistance	111 (7.0)	39 (4.3)	0.0064
Daily life activities			
Ambulatory*	1308 (83.7)	614 (69.1)	<0.0001
Use of wheelchair [outdoor only]	92 (5.9)	85 (9.5)	0.0007
Use of wheelchair [outdoor and indoor]	113 (7.2)	138 (15.4)	<0.0001
Bedridden	48 (3.0)	51 (5.7)	0.0016
Vital signs at presentation			
Systolic blood pressure, mmHg	150 ± 34	139 ± 32	<0.0001
Systolic blood pressure <90 mmHg*	28 (1.7)	41 (4.5)	<0.0001
Heart rate, bpm	97 ± 28	93 ± 25	0.0008
Heart rate <60 bpm*	103 (6.6)	62 (6.9)	0.74
Body temperature>37.5 degree Celsius	51 (3.3)	69 (7.8)	<0.0001
NYHA Class III or IV	1364 (87.2)	777 (86.7)	0.69
Tests at admission			
LVEF	45.0 ± 16.4	48.4 ± 16.7	<0.0001
HFrEF (LVEF < 40%)*	650 (41.5)	290 (32.3)	<0.0001
HFmrEF (LVEF 40–49%)	289 (18.8)	169 (18.8)	0.82
HFpEF (LVEF ≥ 50%)	625 (39.9)	438 (48.3)	<0.0001
BNP, pg/mL	686 (368–1188)	721 (402–1397)	0.028
Serum creatinine, mg/dL	1.06 (0.81–1.48)	1.24 (0.85–1.86)	<0.001
eGFR <30 mL/min/1.73m^2^*	347 (22.1)	304 (33.8)	<0.0001
Blood urea nitrogen, mg/dL	22 (16–30)	23 (18–32)	0.001
Sodium <135 mEq/L*	157 (10.0)	154 (17.5)	<0.0001
Anemia*^§^	899 (57.4)	751 (83.6)	<0.0001
C reactive protein>1 mg/dL	476 (30.7)	504 (56.7)	<0.0001
AST	31 (22–46)	29 (21–45)	0.0750
Cholinesterase	219 (181–267)	161 (127–200)	<0.0001
ACE-I or ARB**	725 (46.2)	378 (42.1)	0.0480
Beta-blocker**	584 (37.2)	350 (39.0)	0.4125
Ca-channel blocker**	589 (37.6)	341 (38.0)	0.8629
Aspirin**	499 (31.8)	287 (32.0)	0.9642
GNRI	98.9 (92.4–105.5)	86.8 (80.7–94.2)	<0.0001
Length of hospital stay	15 [11–23]	18 [12–29]	<0.0001

^*^20 risk-adjusting variables and **5 additional risk-adjusting variables selected for multivariable models.

^||^Body mass index was calculated as weight in kilograms divided by height in meters squared.

^§^Anemia was defined by the World Health Organization criteria (hemoglobin <12.0 g/dL in women and <13.0 g/dL in men).

CONUT = Controlling Nutritional Status; BMI = body mass index; ACS = acute coronary syndrome, HF = heart failure, PCI = percutaneous coronary intervention; CABG = coronary artery bypass graft; BP = blood pressure; bpm = beat per minute; NYHA = New York Heart Association, LVEF = left ventricular ejection fraction; HFrEF = heart failure with reduced ejection fraction; HFmrEF = heart failure with mid-range ejection fraction; HFpEF = heart failure with preserved ejection fraction; BNP = brain-type natriuretic peptide; GFR = estimated glomerular filtration rate, AST = aspartate aminotransferase, ACE-I = angiotensin converting enzyme inhibitor, ARB = angiotensin 2 receptor blocker, GNRI = geriatric nutritional risk index.

As a sensitivity analysis, the patients were grouped into 4 classes on the basis of their CONUT scores. We defined a CONUT score of 0–1 as normal, 2–4 as mild high, 5–8 as moderate high, and 9–12 as marked high according to the previous reports^11,22^. Comparisons among 4 groups were performed using the chi-square test for categorical variables and 1-way ANOVA or Kruskal-Wallis test for continuous variables in addition to the Cochran-Armitage trend test in order to assess the trend across the 4 groups. We used a multivariable logistic regression model including the grades of CONUT score as continuous variables to estimate the incremental impact on the primary outcome measure. In addition, we also evaluated the crude incidence rate of primary outcome measures according to the severity of each component of the CONUT score for the understanding of the incremental impact of the score. We also stratified patients into 2 groups: those with CONUT score>3 and those with CONUT score 0–3 and used the multivariable logistic regression model to investigate the effect of CONUT score>3 on the primary outcome. In supplementary material, we described the methods of additional analyses.

All statistical analyses were conducted by a physician (T.K.) and a statistician (T.M.) using JMP 13.0 or SAS 9.4 (both SAS Institute Inc., Cary, North Carolina). All the reported P values were 2-tailed, and P values <0.05 were considered statistically significant.

## Results

### Baseline characteristics; high versus low CONUT score groups

Patients in the high CONUT score group were older, were more often female, and were more likely to have a smaller body mass index, renal dysfunction (estimated glomerular filtration [eGFR] rate <30 mL/min/1.73m2), malignancy, cognitive dysfunction, low systolic blood pressure at presentation, anemia, hyponatremia, and high CRP levels (>1.0 mg/dL), and had higher BNP levels and lower cholinesterase levels than those in the low CONUT score group (Table 1). On the other hands, patients in the low CONUT score group were more likely to have an ejection fraction <40%, and be currently smoking, ambulatory, and living alone. Patients in the high CONUT score group were less likely to be administered with angiotensin converting enzyme inhibitor or angiotensin 2 receptor blocker before admission than those in the low CONUT score group. Length of hospital stay was longer in the high CONUT score group (Table 1). In Supplementary Table 3, we compared the baseline characteristics of the patients with and without available CONUT scores.

### Clinical outcomes; high versus low CONUT score groups

The primary outcome measure (in-hospital all-cause death) occurred in 69 patients (4.4%) in the low CONUT score group and 81 patients (9.0%) in the high CONUT score group (Fig. 2B and Table 2). After adjusting for confounders, the excess risk of high CONUT score relative to low CONUT score for in-hospital all-cause death remained significant (OR: 1.61, 95% CI: 1.05–2.44, P = 0.027). The adjusted risk for cardiovascular death did not differ significantly between high and low CONUT score groups, while the excess adjusted risk of high CONUT score relative to low CONUT score for non-cardiovascular death was significant. Infection during hospitalization occurred in 200 patients (12.7%) in the low CONUT score group and 197 patients (21.9%) in the high CONUT score group (Table 2). Even after adjusting the confounders, the excess risk of high CONUT score relative to low CONUT score for infection during hospitalization remained significant (OR: 1.66, 95% CI: 1.30–2.12, P < 0.0001). Other cardiovascular outcomes and details were presented in Supplementary Table 4.

**Table 2:** In-hospital Outcomes regarding high versus low CONUT score groups.

	Low CONUT score (N = 1568) N of patients with event (%)	High CONUT score (N = 898) N of patients with event (%)	Crude odds ratio	95%CI	P value	Adjusted odds ratio	95%CI	P value
**Primary outcome**								
All-cause death	69 (4.4)	81 (9.0)	2.18	1.56–3.05	<0.0001	1.61	1.05–2.44	0.027
**Secondary outcome**								
Cardiovascular death	58 (3.7)	50 (5.5)	1.11	1.67–1.83	0.030	1.12	0.68–1.83	0.67
Non-cardiovascular death	11 (0.7)	31 (3.4)	5.06	2.53–10.1	<0.0001	3.67	1.62–8.32	0.0019
Infection during hospitalization	200 (12.7)	197 (21.9)	1.92	1.54–2.38	<0.0001	1.66	1.30–2.12	<0.0001

CONUT = Controlling Nutritional Status; CI = confidence interval.

### Sensitivity analysis

When the patients were classified into 4 groups according to the CONUT score (Fig. 2A and Supplementary Table 5 and 6), the trend of baseline characteristics was fully consistent with the main analysis. The increasing CONUT score grades were associated with incrementally higher risk for in-hospital all-cause death (Fig. 2C) and infection during hospitalization (in-hospital all-cause death: adjusted OR per 1 grade increase: 1.62, 95% CI: 1.24–2.13, P = 0.0004, and infection during hospitalization: adjusted OR per 1 grade increase: 1.54, 95% CI: 1.31–1.80, P < 0.0001). The increasing score grades of each component of CONUT score were also associated with the increasing risk for all-cause death (Fig. 2D and Supplementary Table 7).

When we stratified patients into 2 groups according to CONUT score >3 (N = 1284, 52.1%) and CONUT 0–2 (N = 1182, 47.9%), the trend was generally consistent with that when stratified by the original definition (Supplementary Table 8). The risk for in-hospital death in the CONUT score >3 relative to the CONUT score 0–2 remained significant (adjusted OR: 1.75, 95% CI: 1.11–2.77, P = 0.015).

### Subgroup analysis for the primary outcome measure and infection during hospitalization; high versus low CONUT score groups

**Figure 3:**
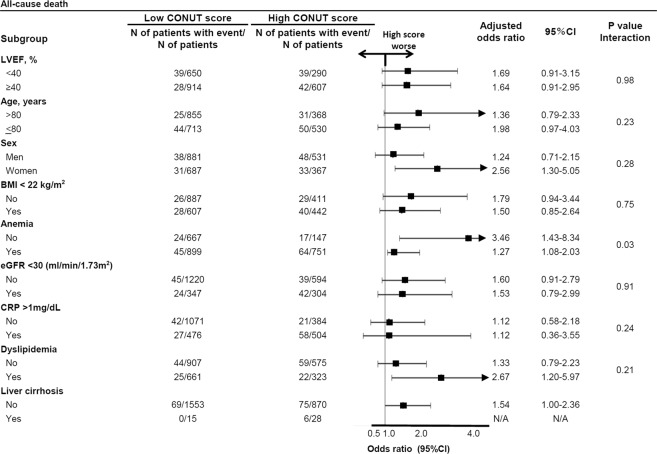
Subgroup analysis for the effect of the high versus low CONUT score on the primary outcome measure (in-hospital death). CONUT = Controlling Nutritional Status, CI = confidence interval, LVEF = left ventricular ejection fraction, BMI = body mass index, eGFR=estimated glomerular filtration rate, CRP = C reactive protein, N/A = not available.

**Figure 4:**
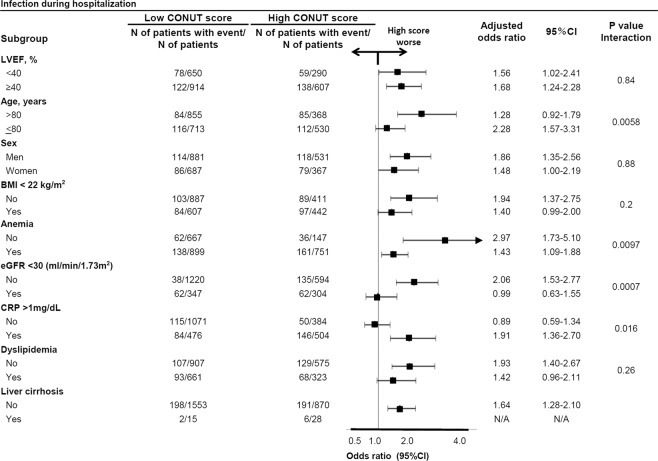
Subgroup analysis for the effect of the high versus low CONUT score on the infection during hospitalization. CONUT = Controlling Nutritional Status, CI = confidence interval, LVEF = left ventricular ejection fraction, BMI = body mass index, eGFR=estimated glomerular filtration rate, CRP = C reactive protein.

In the subgroup analyses stratified by LVEF, age, sex, BMI, anemia, renal dysfunction, elevated CRP levels, and the presence of dyslipidemia, there were no significant interactions between the subgroup factors and the effect of high CONUT score relative to low CONUT score for in-hospital all-cause death, except for anemia (Fig. 3). Patients without anemia showed directionally worse impact of the high CONUT score over the low CONUT score for the in-hospital mortality (Fig. 3). There were 4 significant high score-by-subgroup interactions for infection during hospitalization: patients less than 80 years old, those without anemia, and those without renal dysfunction, and those with CRP levels> 1 mg/dL showed directionally worse impact of the high CONUT score (Fig. 4).

### Association of CRP levels at admission and GNRI with CONUT scores

There was a positive but mild correlation with CRP levels at admission and CONUT scores (Supplementary Fig. 1). We also calculated GNRI from serum albumin levels and body weight and height. GNRI was inversely correlated with the increase of CONUT score (Supplementary Fig. 2). The prognostic accuracy did not differ significantly between CONUT score and GNRI (Supplementary Fig. 3).

## Discussions

The principal findings in the present study were as follows; (1) the risk of high CONUT score relative to low score for in-hospital mortality was significant even after adjusting for confounders; (2) the excess adjusted risks for non-cardiovascular death and infection during hospitalization were also significant in patients with high CONUT score; and (3) the interaction between the subgroup factors and the effect of high CONUT score was observed in patients with anemia for all-cause death and age over 80, anemia, renal dysfunction and high CRP levels (>1 mg/dL) at presentation for infection during hospitalization.

There have been many reports about the association between each component of CONUT score and outcomes. The drop in the lymphocyte count which is caused by the physical stress, malnutrition and chronic inflammation^23,24^ predicts the one-year outcome in patients with advanced HF^25^ or coronary artery disease^26^. Hypoalbuminemia is well known predictor for the short-term prognosis in patients with ADHF^27,28^, although albumin level is influenced by hemodilution, renal loss, and shortened half-life due to severe illness. In the present analysis, cholinesterase, a rapid turnover protein, decreased in a step-wised manner as the CONUT score increased. Cholesterol level, a well responder to dietary intake^29^, predicts in-hospital mortality in patients with ADHF^30^. These underlying mechanisms were closely linked to not only the nutrition but also to the acute exacerbation of the disease; therefore, the CONUT score, a complex of immunity status, protein reserve, and lipid metabolism, were hypothesized to have a significant impact on ADHF patients incrementally. CONUT score was originally designed to predict “acute worsening” in surgical patients [11,22] and, thereafter, adapted to chronic heart failure^4,12–14^; this fact may justify its applicability in patients with ADHF. In the present study, we showed that the CONUT score on admission successfully stratified the risk for in-hospital mortality and hospital-acquired or evident infection rate with ADHF, and their effect was in an incremental manner. The link between CONUT score at discharge and the long-term outcomes were reported by Yoshihisa *et al*.^22^ and the link between CONUT score on admission and the long-term outcomes were reported by Iwakami *et al*.^31^. Shirakabe *et al*. also reported that the CONUT score on admission was linked to in-hospital mortality^32^; however, due to the lack of power in limited numbers of enrollment, the analyses of in-hospital mortality with adjustment for confounders was not fully performed in either study^31,32^. The CONUT score on admission well represented a vicious cycle in ADHF where HF caused the malnutrition through fluid retention, and malnutrition, in turn, leads to inflammation and neurohormonal activation.

CONUT score was closely associated with monocyte TNF-α production in chronic heart failure^13^. Although we did not examine the concentration of TNF-α nor long-term outcomes, the correlation between CONUT score and inflammation was also shown in the present study as well as those with chronic heart failure. GNRI, another important risk score which included serum albumin and body weight, was also correlated to CONUT scores and had an equivalent risk prediction ability. Because the number of patients with liver cirrhosis was relatively small in the present study and we have included it in multivariable analyses, liver cirrhosis has not dramatically influenced the results of the study. However, it is very important to investigate the potential presence of liver dysfunction by blood tests^33^ and imaging modalities such as computed tomography, abdominal ultrasound, and transient elastography^34^ when the CONUT score of a given patient with ADHF is high. The onset of liver cirrhosis is characterized by low albumin and platelet levels and spleen enlargement. In addition, liver congestion is a hallmark of congestive heart failure^35^ with an increased inflammation^36^.

The in-hospital management of patients with ADHF is very important in medical practice and from the social and economic viewpoint^37^. Hospital-acquired infection is one of the major causes of hospital-associated disability^38^. Thus, we set newly-acquired or emerging infection after admission as one of the secondary endpoints. Interaction analyses highlighted the useful situation for assessing the CONUT score for infection in patients without anemia and renal dysfunction, those with high CRP levels, relatively younger patients with age <80 years.

### Limitations

This study has several limitations. First, the diagnosis of infection was based on the physician’s judgment. In addition, due to the complex nature of the infection, we could not differentiate between newly-acquired infections during hospitalization and the infections that became obvious days after the admission. Second, we did not collect data regarding statin use. However, according to the definition of dyslipidemia as receiving anti-dyslipidemia drugs or having a total cholesterol level ≥220 mg/dL, we could perform the subgroup analyses and showed no interaction between the dyslipidemia and the effect of high CONUT score on in-hospital mortality or infection. Third, laboratory abnormalities of liver function tests such as cholinesterase were not included in the multivariable model due to multicollinearity because the CONUT score was derived from albumin levels, one of the liver function tests. Fourth, there remain unmeasured confounders affecting the in-hospital prognosis, although we conducted extensive statistical adjustment for the measured confounders. Fifth, several subgroup analyses have a risk for multiple comparison as well as small sample size with low statistical power. Finally, those excluded for missing data included patients with women, no anemia, and no ACS.

## Conclusions

High CONUT score was independently associated with higher risk for in-hospital mortality and infection in an incremental manner in patients hospitalized for ADHF. CONUT score can play a role in identifying those patients who need to be monitored carefully for infections at the time of hospitalization for ADHF.

## Supplementary information

Supplementary materials.

## Data Availability

The datasets generated during and/or analyzed during the current study are available from the corresponding author on reasonable request.
