# Supplementary material for: Association with Controlling Nutritional Status (CONUT) Score and In-hospital Mortality and Infection in Acute Heart Failure
Source: Sci Rep. 2020 Feb 24;10:3320. doi: 10.1038/s41598-020-60404-9 (PMC7039945; doi:10.1038/s41598-020-60404-9)
Supplement: Supplementary file 1 — Supplementary materials. [file 41598_2020_60404_MOESM1_ESM.pdf]

SUPPLEMENTARY MATERIALS

for:

Association with Controlling Nutritional Status (CONUT) Score and In-hospital Mortality and Infection in Acute Heart Failure

Takao Kato, Hidenori Yaku, Takeshi Morimoto, Yasutaka Inuzuka, Yodo Tamaki, Erika Yamamoto, Yusuke Yoshikawa, Takeshi Kitai, Ryoji Taniguchi, Moritake Iguchi, Masashi Kato, Mamoru Takahashi, Toshikazu Jinnai, Tomoyuki Ikeda, Kazuya Nagao, Takafumi Kawai, Akihiro Komasa, Ryusuke Nishikawa, Yuichi Kawase, Takashi Morinaga, Kanae Su, Mitsunori Kawato, Yuta Seko, Moriaki Inoko, Mamoru Toyofuku, Yutaka Furukawa, Yoshihisa Nakagawa, Kenji Ando, Kazushige Kadota, Satoshi Shizuta, Koh Ono, Yukihito Sato, Koichiro Kuwahara, Neiko Ozasa, Takeshi Kimura.

contain

Supplementary methods

8 Supplementary Tables and

3 Supplementary FIgures

Supplementary Methods

Ethics

The local ethical committees of National Hospital Organization Kyoto Medical Center, Mitsubishi Kyoto Hospital, Shimabara Hospital, Daini Okamoto General Hospital, Japanese Red Cross Otsu Hospital, Shiga Medical Center for Adults, Hikone Municipal Hospital, Osaka Red Cross Hospital, Kitano Hospital, Kishiwada City Hospital, Kansai Electric Power Hospital, Tenri Hospital, Hyogo Prefectural Amagasaki General Medical Center, Kobe City Medical Center General Hospital, Japanese Red Cross Wakayama Medical Center, Shizuoka General Hospital, Kurashiki Central Hospital, an Kokura Memorial Hospital approved this study and waived the requirement for written informed consent.

Definitions for the baseline factors

The definitions of the baseline factors in KCHF registry were according to the previous repot [1]. Atrial fibrillation (AF) included paroxysmal AF, persistent AF, and permanent AF. Hypertension was defined as receiving anti-hypertensive drugs or systolic blood pressure ≥140 mmHg or diastolic blood pressure ≥90 mmHg. Diabetes mellitus was defined as treatment with oral hypoglycemic agents and/or insulin, prior clinical diagnosis of diabetes, glycated hemoglobin level ≥6.5 %, casual blood glucose level ≥200 mg/dl, or fasting blood glucose level ≥126 mg/dl. The presence of COPD was determined clinically by local investigators, based on history, clinical presentation, previous examinations, and medications, recorded as COPD in the case report form at

enrolment. Poor medical adherence was judged by the attending physician. Public assistance is one of social security systems in Japan, which are explained elsewhere (http://www.ipss.go.jp/s-info/e/ssj2014/006.html). Underlying heart disease was defined as the most likely cause of structural or functional cardiac disorders, among those only one category was chosen. The underlying heart disease was classified as (i) coronary artery disease, (ii) hypertensive heart disease, (iii) cardiomyopathy, (iv) valvular heart disease, (v) other heart diseases. Coronary artery disease was defined as acute coronary syndrome (ACS), old myocardial infarction, or prior PCI/CABG. ACS was defined as the range of myocardial ischemic states that includes ST-elevated myocardial infarction, non-ST elevated myocardial infarction, or unstable angina. Primary cardiomyopathy was classified as hypertrophic cardiomyopathy, dilated cardiomyopathy, and dilated phase of hypertrophic cardiomyopathy. Valvular heart disease was classified as moderate to severe aortic stenosis, aortic regurgitation, mitral stenosis, mitral regurgitation (excluding functional mitral regurgitation), tricuspid regurgitation, and prosthetic valve dysfunction. As the valvular heart disease, we chose only one category, which seemed to have most closely related to acute heart failure. Other heart diseases included other cardiomyopathy, arrhythmia (bradycardia or tachycardia), congenital heart disease, and constrictive pericarditis. Other cardiomyopathy included arrhythmogenic right ventricular dysplasia, takotsubo cardiomyopathy, cardiac sarcoidosis, cardiac amyloidosis, left ventricular noncompaction, drug-induced cardiomyopathy, pacemaker induced cardiomyopathy, mitochondrial cardiomyopathy, peripartum cardiomyopathy, alcoholic cardiomyopathy, beriberi heart, and others.

Chronic kidney disease was defined as an estimated glomerular filtration rate (eGFR) <60 mL/min per 1.73 m^2 at admission. The eGFR was calculated using the equation for the Japanese population: eGFR = 194 × (serum creatinine$^{-1.094}$) × (age$^{-0.287}$) × 0.739 (for women). Geriatric nutritional risk index was calculated as follows: 14.89 × (serum albumin[g/dL]) + 41.7 × (body weight/ ideal body weight). Ideal body weight was calculated as follows: 22 ×(height[m]^2).

Definitions for the causes of death and in-hospital adverse events

The definitions of the causes of death and in-hospital adverse events in KCHF registry were according to the previous repot [1]. The causes of death were adjudicated by a clinical event committee. Every death was placed into 1 of the 2 categories in the KCHF registry: 1) cardiovascular death, which includes death related to heart failure (HF), sudden death, death related to stroke, and other cardiovascular death; and 2) non-cardiovascular death, which includes pulmonary disease, sepsis, other infection, gastrointestinal disease, malignancy, renal failure, and other non-cardiovascular death. Sudden death was defined as unexplained death in a previously stable patient. Stroke was defined as ischemic or hemorrhagic stroke requiring or prolonging hospitalization with symptoms lasting >24 hours. Infection included bacterial pneumonia, acute cholangitis, peritonitis, purulent arthritis, and other infection except for pulmonary infection.

Statics for additional analyses

The relationship between two variables is presented on a scattered plot figure. The Pearson correlation coefficient was calculated and presented. Using CONUT score and GNRI respectively,

we showed receiver-operating curves with an area under the curve (AUC) and provided the sensitivity and 1-specificity to predict the in-hospital deaths. We compared the AUCs of the two models using JMP 13.0 (SAS Institute Inc., Cary, North Carolina).

References

1. Yaku H, Ozasa N, Morimoto T, Inuzuka Y, Tamaki Y, Yamamoto E, Yoshikawa Y, Kitai T, Taniguchi R, Iguchi M, Kato M, Takahashi M, Jinnai T, Ikeda T, Nagao K, Kawai T, Komasa A, Nishikawa R, Kawase Y, Morinaga T, Su K, Kawato M, Sasaki K, Toyofuku M, Furukawa Y, Nakagawa Y, Ando K, Kadota K, Shizuta S, Ono K, Sato Y, Kuwahara K, Kato T, Kimura T, on behalf of the KCHF Study Investigators. Demographics, Management, and In-hospital Outcomes of Patients with Hospitalized Acute Heart Failure Syndromes in the Contemporary Real Clinical Practice in Japan: Observations from the Prospective, Multicenter Kyoto Congestive Heart Failure (KCHF) registry Circ J. 2018 Sep 26. doi: 10.1253/circj.CJ-17-1386.

Supplementary Table 1. Numbers of patients with missing values for the calculation of CONUT score

**Variables**	**Numbers of patients with missing values**
Albumin	108
Total cholesterol	1,142
Lymphocyte count	825
Unavailable CONUT score	1,590

CONUT = Controlling Nutritional Status.

Supplementary Table 2. Individual components of the CONUT score

Lymphocyte (/µL)	Ly ≥1600	1200≤ Ly <1600	800≤ Ly <1200	Ly <800
**Scoring**	0	1	2	3
**%**	28.9%	20.1%	26.3%	24.1%

Total cholesterol (mg/dL)	TC ≥180	140≤ TC <180	100≤ TC <140	TC <100
**Scoring**	0	1	2	3
**%**	26.1%	39.9%	29.0%	4.7%

Albumin (g/dL)	Alb ≥3.5	3≤ Alb <3.5	2.5≤ Alb <3	Alb <2.5
**Scoring**	0	2	4	6
**%**	53.1%	30.9%	12.2%	3.6%

Ly = lymphocyte count, TC = total cholesterol, Alb = albumin.

Supplementary Table 3. Baseline characteristics of the patients with versus without CONUT score data

Variables	Available CONUT score data (N= 2466)	Unavailable CONUT score data (N= 1590)	P value
Age	81 [71-87]	80 [72-86]	0.10
Men	1330 (57.2)	913 (52.4)	0.0024
BMI < 22 kg/m^2	1002 (45.3)	786 (48.3)	0.09
ACS	153 (6.5)	85 (4.8)	0.022
Prior HF	834 (36.3)	614 (36.3)	0.99
Hypertension	1664 (71.6)	1249 (71.7)	0.92
Diabetes	850 (36.5)	625 (35.9)	0.66
COPD	388 (16.7)	280 (16.0)	0.60
Prior MI	530 (22.8)	378 (21.7)	0.41
Prior stroke	388 (16.7)	280 (16.0)	0.60
Malignancy	340 (14.6)	247 (14.2)	0.69
Current smoking	281 (12.1)	193 (11.1)	0.96
Systolic BP < 100mmHg	208 (8.9)	123 (7.0)	0.030
HR < 60 bpm	143 (6.1)	101 (5.8)	0.64
AF at presentation	847 (36.4)	607 (34.8)	0.30
eGFR<30mL/min/1.73m^2	621 (26.7)	499 (28.8)	0.14
Na < 135 mEq/L	303 (13.0)	218 (12.6)	0.68
Anemia	1596 (68.7)	1114 (64.4)	0.0027
LVEF < 40%	878 (37.8)	678 (39.1)	0.40
Ambulatory	1792 (77.1)	1360 (79.3)	0.21
Living alone	504 (21.8)	359 (20.8)	0.47

CONUT = Controlling Nutritional Status; BMI = body mass index; ACS = acute coronary syndrome, HF = heart failure; COPD = chronic obstructive pulmonary disease; MI = myocardial infarction; BP = blood pressure; HR = heart rate; AF = atrial fibrillation/flutter; eGFR = estimated glomerular filtration rate; LVEF = left ventricular ejection fraction.

Supplementary Table 4. Details of in-hospital cardiovascular outcomes

	Low CONUT score (N=1568) N of patients with event (%)	High CONUT score (N=898) N of patients with event (%)	Crude odds ratio	95%CI	P value
**Details of cardiovascular death**					
Death from heart failure	50 (3.2)	38 (4.2)	1.34	0.87-2.06	0.18
Sudden death	3 (0.2)	5 (0.6)	2.92	0.70-12.25	0.15
Other cardiac death	0 (0.0)	5 (0.6)	N/A	N/A-	0.0064
Vascular death	5 (0.3)	2 (0.2)	0.70	0.14-3.60	1.0000
**Cerebral infarction**	17 (1.1)	23 (2.6)	2.40	1.27-4.51	0.0074
**Cerebral hemorrhage**	6 (0.4)	7 (0.8)	2.05	0.69-6.10	0.25

CONUT = Controlling Nutritional Status; CI = confidence interval.

Supplementary Table 5. Patient characteristics in the 4 subgroups according to the CONUT score

Variables	Normal CONUT score (N=430, 17.4%)	Mild high CONUT score (N=1138, 46.1%)	Moderate high CONUT score (N=774, 31.3%)	Marked high CONUT score (N=124, 5.0%)	P value	Cochran-Armitage trend test
Age	75 [66-83]	79 [72-86]	82 [74-87]	82 [77-87]	<0.0001	
Age >80 years	156 (36.2)	557 (48.9)	449 (58.0)	81 (65.3)	<0.001	<0.0001
Male	251 (58.3)	630 (55.3)	453 (58.5)	78 (62.9)	0.25	0.33
BMI <22 kg/m^2	144 (6.1)	463 (42.7)	387 (52.8)	55 (47.0)	<0.0001	<0.0001
ACS as etiology	38 (8.8)	82 (7.2)	46 (5.9)	4 (3.2)	0.094	0.013
Prior hospitalization for HF	114 (27.0)	419 (37.1)	291 (38.0)	47 (38.2)	0.0007	0.0012
Hypertension	300 (69.7)	848 (74.5)	538 (69.5)	78 (62.9)	0.0084	0.10
Diabetes mellitus	151 (35.1)	419 (36.8)	285 (36.8)	48 (38.7	0.87	0.49

AF/AFL	139 (32.3)	476 (41.8)	337 (43.5)	57 (45.9)	0.0007	0.0003
Prior myocardial infarction	94 (21.8)	273 (23.9)	164 (21.1)	28 (22.5)	0.52	0.62
Prior stroke	67 (15.5)	187 (16.4)	135 (17.4)	15 (12.)	0.45	0.94
Chronic lung disease	26 (6.0)	107 (9.4)	73 (9.4)	10 (8.0)	0.16	0.17
Liver cirrhosis	3 (0.7)	12 (1.1)	20 (2.6)	8 (6.5)	<0.0001	<0.0001
Current smoker	84 (19.5)	149 (13.1)	73 (9.4)	14 (11.2)	<0.0001	<0.0001
Ambulatory	379 (88.5)	928 (81.9)	531 (69.4)	83 (67.4)	<0.0001	<0.0001
Living alone	94 (22.0)	273 (24.0)	145 (18.8)	27 (21.7)	0.0063	0.13
Ejection fraction <40%	187 (43.6)	463 (40.7)	250 (32.3)	40 (32.5)	<0.0001	<0.0001
Systolic BP <90 mmHg	9 (2.0)	19 (1.6)	35 (4.5)	6 (4.8)	0.0009	0.0010
Heart rate <60 bpm	31 (7.2)	72 ‘6.3)	55 (7.1)	7 (5.7)	0.84	0.88
Anemia	190 (44.3)	709 (62.3)	637 (82.3)	114 (91.9)	<0.0001	<0.0001

eGFR <30 mL/min/1.73m^2	72 (16.7)	275 (24.1)	265 (34.2)	39 (31.4)	<0.0001	<0.0001
Na <135 mEq/L	31 (7.2)	126 (11.0)	128 (16.5)	26 (20.9)	<0.0001	<0.0001
AST	32 (23-45)	30 (22-47)	29 (21-45)	32 (23-50)	0.083	
Cholinesterase	254 (211-304)	209 (174-250)	167 (134-205)	119 (94+149)	<0.0001	
ACE-I or ARB	180 (41.9)	545 (47.9)	331 (42.8)	47 (37.9)	0.022	0.31
Beta-blocker	140 (32.6)	444 (39.0)	303 (39.2)	47 (37.9)	0.096	0.086
Ca-channel blocker	144 (33.5)	445 (39.1)	296 (38.2)	45 (36.3)	0.22	0.32
Aspirin	120 (27.9)	379 (33.3)	253 (32.7)	34 (27.4)	0.13	0.50
GNRI	102.4 (96.9-108.6)	97.2 (90.8-104.1)	87.8 (82.0-95.2)	80.2 (74.1-84.9)	<0.0001	

CONUT = Controlling Nutritional Status; BMI = body mass index; ACS = acute coronary syndrome, HF = heart failure, AF/AFL = atrial fibrillation/ atrial flutter; BP = blood pressure; bpm = beat per minute; eGFR = estimated glomerular filtration rate; AST= aspartate aminotransferase; ACE-I=angiotensin converting enzyme inhibitor; ARB=angiotensin 2 receptor blocker; GNRI=geriatric nutritional risk index.

Supplementary Table 6. In-hospital outcomes in the 4 subgroups according to the CONUT score

Variables	Normal CONUT score (N=430)	Mild high CONUT score (N=1138)	Moderate high CONUT score (N=774)	Marked high CONUT score (N=124)	P value	Cochran-Armitage trend test
All cause death	13 (3.0)	56 (4.9)	56 (7.2)	25 (20.1)	<0.0001	<0.0001
Cardiovascular death	12 (2.7)	46 (4.0)	34 (4.3)	16 (12.9)	<0.0001	0.0005
Non-cardiovascular death	1 (0.2)	10 (0.8)	22 (2.8)	9 (7.2)	<0.0001	<0.0001
Infection during hospitalization	35 (8.1)	165 (14.5)	157 (20.2)	40 (32.2)	<0.0001	<0.0001

CONUT = Controlling Nutritional Status.

Supplementary Table 7. In-hospital all-cause death in each component of CONUT scores

**Lymphocyte (/μL)**	**Ly ≥1600**	**1200≤ Ly <1600**	**800≤ Ly <1200**	**Ly <800**		
**Scoring**	**0**	**1**	**2**	**3**		
	N of events/ No patients (%)	N of events/ No patients (%)	N of events/ No patients (%)	N of events/ No patients (%)	P value	Cochran-Armitage trend test
All-cause death	27/715 (3.7)	23/505 (4.5)	42/650 (6.4)	58/596 (9.7)	<0.0001	<0.0001
Total cholesterol (mg/dL)	TC ≥180	140≤ TC <180	100≤ TC <140	TC <100		
Scoring	0	1	2	3		
	N of events/ No patients (%)	N of events/ No patients (%)	N of events/ No patients (%)	N of events/ No patients (%)	P value	Cochran-Armitage trend test
All-cause death	30/646 (4.6)	49/986 (4.9)	57/717 (7.9)	14/117 (11)	0.0012	0.0003
Albumin (g/dL)	Alb ≥3.5	3≤ Alb <3.5	2.5≤ Alb <3	Alb <2.5		
Scoring	0	2	4	6		
	N of events/ No patients (%)	N of events/ No patients (%)	N of events/ No patients (%)	N of events/ No patients (%)	P value	Cochran-Armitage trend test
All-cause death	61/1310 (4.6)	47/764 (6.1)	26/301 (8.6)	16/91 (17)	<0.0001	<0.0001

Ly = lymphocyte count, TC = total cholesterol, Alb = albumin.

Supplementary Table 8. Patient Characteristics of those with CONUT score >3 and those with CONUT score 0-3.

Variables	CONUT score		
	CONUT score 0-3 (N=1182, 47.9%)	CONUT score >3 (N=1284, 52.1%)	P value
Age, years*	78 [69-84]	81 [74-87]	<0.0001
Age >80 years	508 (43.0)	735 (57.2)	<0.0001
Men*	670 (56.7)	742 (57.8)	0.5964
BMI<22 kg/m^2*	434 (38.4)	615 (50.5)	<0.0001
ACS*	90 (7.6)	80 (6.2)	0.17
Prior hospitalization for HF*	389 (33.3)	482 (38.0)	0.017
Atrial fibrillation or flutter*	449 (38.0)	560 (43.6)	0.0047
Hypertension*	861 (72.8)	903 (70.3)	0.18
Diabetes mellitus*	421 (35.6)	482 (37.5)	0.33
Dyslipidemia	512 (43.3)	472 (36.8)	0.001
Prior myocardial infarction*	275 (23.3)	284 (22.1)	0.50
Prior stroke*	185 (15.7)	219 (17.1)	0.35
Current smoking*	179 (15.1)	141 (11.0)	0.0027
Chronic lung disease*	106 (9.0)	110 (8.6)	0.77
Liver cirrhosis**	11 (0.9)	32 (2.5)	0.0032
Living alone*	291 (24.7)	248 (19.4)	0.0015
Ambulatory*	1012 (86.1)	909 (71.5)	<0.0001
Systolic blood pressure <90 mmHg*	22 (1.9)	47 (3.7)	0.0069
Heart rate <60 bpm*	77 (6.6)	88 (6.9)	0.74
HFrEF (LVEF <40%)*	506 (43.0)	434 (33.8)	<0.0001
eGFR <30 mL/min/1.73m^2*	231 (19.6)	420 (32.7)	<0.0001
Sodium <135 mEq/L*	110 (9.3)	201 (15.7)	<0.0001
Anemia*	609 (51.6)	1041 (81.1)	<0.0001
C reactive protein >1 mg/dL	340 (29.2)	640 (50.4)	<0.0001
AST	31 [23-46]	29 [21-46]	0.064
Cholinesterase	228 [191-278]	170 [133-211]	<0.0001
ACE-I or ARB**	548 (46.4)	555 (43.2)	0.12
Beta-blocker**	450 (38.1)	484 (37.7)	0.86
Ca-channel blocker**	462 (39.1)	468 (36.5)	0.18
Aspirin**	366 (31.0)	420 (32.7)	0.36
GNRI	100.2 [94.0-106.7]	88.7 [82.4-96.9]	<0.0001

*20 risk-adjusting variables and **5 additional risk-adjusting variables selected for multivariable models.

Supplementary figure legends

Supplementary figure 1. Scattered plot of CONUT score and CRP levels.

Supplementary figure 2. Scattered plot of CONUT score and geriatric nutritional risk index (GNRI)

Supplementary figure 3. Comparison of AUCs of CONUT score and GNRI. AUC=area under the curve.

Supplementary figure 1.

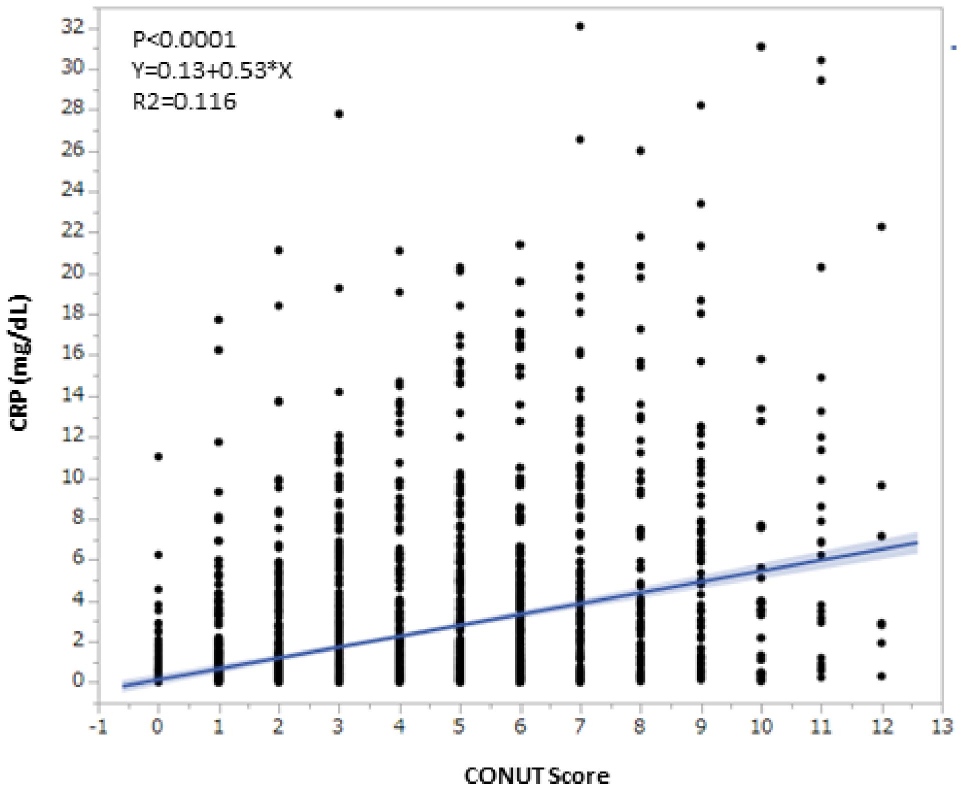

Supplementary figure 2.

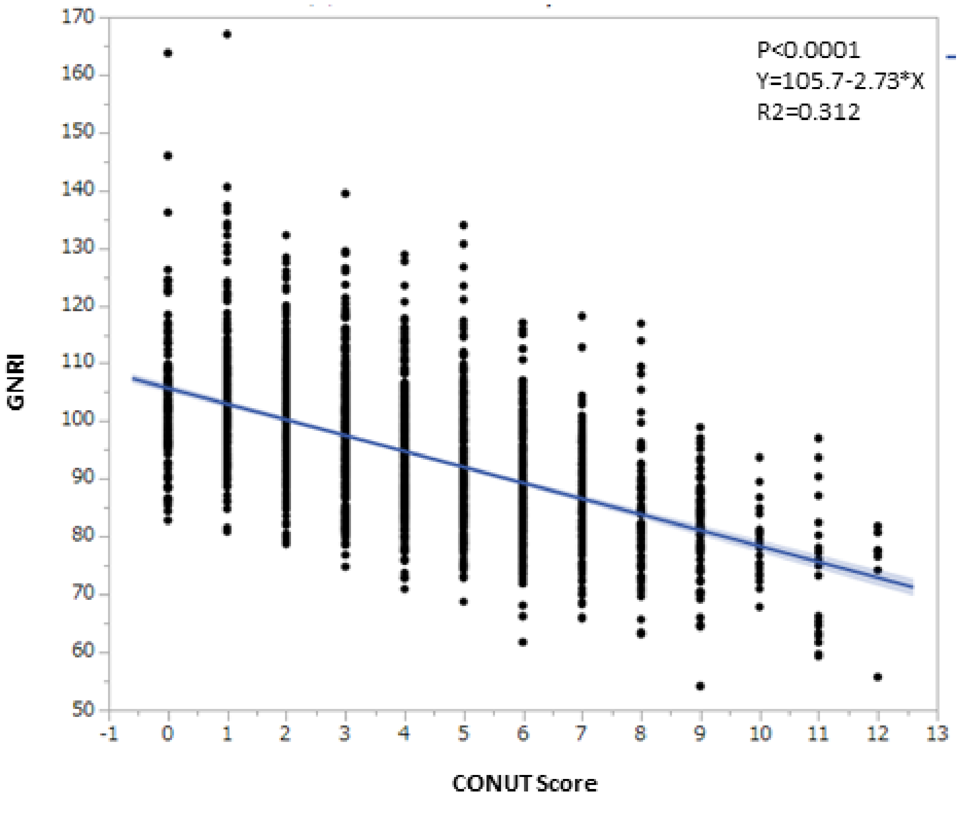

Supplementary figure 3.

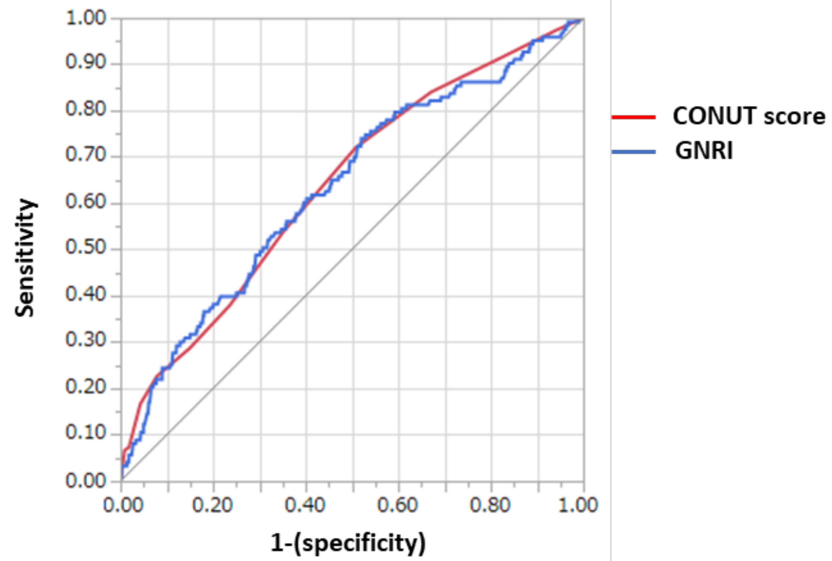

Predictor	Area under the curve	Lower 95%CI	Upper 95%CI
CONUT score	0.640	0.593	0.684
GNRI	0.634	0.581	0.685

Comparison	Difference of AUC	Lower 95%CI	Upper 95%CI	P value
CONUT score vs. GNRI	0.0055	-0.033	0.044	0.78
